# Multi-ancestry genome-wide association meta-analysis identifies candidate genes for computed tomography-based carcass composition traits in pigs

**DOI:** 10.1186/s12711-025-01023-8

**Published:** 2025-12-17

**Authors:** He Han, Pengfei Yu, Zhenyang Zhang, Ran Wei, Wei Zhao, Xiaoliang Hou, Jianlan Wang, Yongqi He, Yan Fu, Zhen Wang, Yuchun Pan, Qishan Wang, Zhe Zhang

**Affiliations:** 1 Zhejiang Key Laboratory of nutrition and breeding for high-quality animal products, College of Animal Sciences, Zhejiang University, Hangzhou, 310058 China; 2 Key Laboratory of Livestock and Poultry Resources Evaluation and Utilization, Ministry of Agriculture and Rural Affairs, Hangzhou, 310030 China; 3 SciGene Biotechnology Co., Ltd., Hefei, 230022 China; 4 Hainan Institute, Zhejiang University, Yongyou Industrial Park, Yazhou Bay Sci-Tech City, Sanya, 572000 China

## Abstract

**Background:**

Carcass composition traits, such as lean meat percentage, bone percentage, and number of ribs, are critical factors determining meat production and profitability of pigs. Traditional slaughter measurements are time-consuming, labor-intensive and invasive and cannot be evaluated on selection candidates. However, computed tomography scanning, a non-invasive technique, enables in vivo measurement of these traits, facilitating rapid accumulation of extensive phenotypic data. Despite these advances, the genetic mechanisms underlying computed tomography-based carcass traits remain largely unexplored.

**Results:**

In this study, we performed a multi-ancestry genome-wide association meta-analysis (MA-GWAMA) using low-coverage whole-genome sequencing data from four breeds (1222 Duroc, 582 Landrace, 1018 Yorkshire, and 448 Piétrain). In total, we identified 11 independent genome-wide significant loci associated with carcass composition traits in the meta-analysis. Compared to standard genomic best linear unbiased prediction, weighting MA-GWAMA-significant SNPs increased genomic prediction accuracy in an independent population (*N* = 365, including 136 Duroc, 65 Landrace, 50 Piétrain, and 114 Yorkshire) by 16.3% for lean meat percentage, by 6.1% for bone percentage, and by 79.4% for number of ribs. Integrating MA-GWAMA results with public eQTL and single-cell data prioritized *ALPK2* as a candidate gene for lean meat percentage, and *ABCD4* and *SLC8A3* as candidate genes for the number of ribs.

**Conclusions:**

Our study demonstrates the efficacy of computed tomography phenotyping coupled with multi-omics integration for dissecting the genetic architecture of porcine carcass composition traits. The prioritized variants and genes provide valuable targets for molecular breeding programs to enhance meat quality in pigs.

**Supplementary Information:**

The online version contains supplementary material available at 10.1186/s12711-025-01023-8.

## Background

Pork is one of the most important sources of meat for humans, accounting for 35% of global meat production in 2024 [1]. Carcass composition traits, such as lean meat percentage (LMP), bone percentage (BP), and number of ribs (NR), are indicative of both pork quality and quantity [2]. Over the past few decades, most pig breeding programs have focused on increasing lean meat content, reducing carcass fat content, and selecting pigs with more ribs. In animal breeding, carcass composition traits can be measured via in vivo scanning techniques, such as ultrasound (A- or B-mode ultrasonography) and computed tomography (CT). Compared to CT, ultrasound is less expensive and easier to operate. However, CT provides more accurate measurements of traits like LMP [3] and is recognized as the “gold standard” to measure carcass composition in the livestock industry [4]. Since 2000, CT scanning has become increasingly prevalent in animal breeding, especially for carcass-related traits. For instance, Prieto et al. [5] utilized CT scanning to accurately predict beef cut composition, intramuscular fat content, and fatty acid profiles at a lower cost than dissection, chemical analysis, and other methods. Aasmundstad et al. [6] developed a method for scoring osteochondrosis in pigs by using information from CT; they also used CT to estimate the genetic parameters of in vivo primal cuts in breeding pigs [7]. To investigate the impact of various diets on changes in the carcass composition of growing-fattening piglets, Lambe et al. [8] used both CT scanning and slaughter measurements and reported high concordance between the two methods. In pig breeding, Picouet et al. [9] employed non constant density estimation to improve the accuracy of predicting pig carcass weight and LMP without prior segmentation of the images.

Despite its potential, CT scanning faces several technical challenges in deriving accurate and scalable phenotypic measurements. These include automated and precise removal of internal organs, accurate segmentation of carcass components such as muscle and fat, and reliable identification of anatomical landmarks necessary for trait quantification. Bardera et al. [10] developed a segmentation approach to remove internal organs from in vivo CT scans of pigs. The method begins by generating a 3D distance map that measures the proximity of internal structures to the body surface. Morphological operations, such as erosion and dilation, are then applied to progressively eliminate internal organs while preserving key anatomical components like muscle, fat, and bone. Xiberta et al. [11] proposed semiautomatic and automatic segmentation algorithms to remove internal organs. The development of deep learning algorithms for image recognition has led to methods for predicting carcass composition trait measurements from CT images. For instance, Pan et al. [12] presented a non-invasive method to automatically classify and quantify the body composition of live pigs using CT imaging and deep neural networks, accurately estimating the proportions of lean meat, fat, and bone. However, the genetic underpinnings of CT-derived traits are poorly understood.

Since the early 2000s, genome-wide association studies (GWASs) have successfully elucidated the genetic mechanisms of complex traits, including carcass composition traits, in various pig populations [13–15]. For example, Zhou et al. [16] performed a GWAS and meta-analysis for LMP in two Duroc pig populations, but the phenotypes for LMP in their study were predicted by measurements of backfat thickness and loin muscle depth instead of actual measurements. Van Son et al. [17] conducted a GWAS of backfat thickness and osteochondrosis in Landrace pigs via data obtained from CT images and fine-mapped pleiotropic genomic regions. Additionally, most pig GWASs to date have used medium-density single nucleotide polymorphism (SNP) chips [18, 19]. With the decreasing cost of whole-genome sequencing (WGS) and improvements in imputation methods, the genetic mechanisms of pig traits are now being uncovered at the WGS level and novel trait-associated loci have been identified [20]. This opens the possibility to explore carcass composition traits in greater detail via SNPs identified through WGS.

Most genetic variants identified by GWAS are located in non-coding regions of the genome [21]. These variants may play a role in regulating gene expression. Transcriptome-wide association studies (TWASs) can combine gene expression measurements with summary statistics from large-scale GWASs to identify significant gene expression-tissue-trait associations [22]. Therefore, the systematic integration of functionally annotated data, such as from the PigGTEx resources [23], allows prioritization of causal variants, genes, pathways, and tissues for carcass composition traits to further elucidate their genetic mechanisms. In addition, with the increasing availability of single-cell RNA sequencing (scRNA-seq) data from various tissues, it becomes increasingly feasible to explore gene-phenotype interrelationships at the cellular level. By correlating these cellular data with GWAS findings, we can identify specific cell types that may be causally linked to complex traits [24].

This study investigated the genetic mechanisms underlying pig carcass composition traits quantified via in vivo CT scanning across four breeds: Duroc, Landrace, Yorkshire, and Piétrain. We estimated the genetic parameters of carcass composition traits and identified candidate genes using a multi-ancestry genome-wide association meta-analysis (MA-GWAMA) based on whole-genome sequencing data. Furthermore, we integrated gene expression data from PigGTEx and publicly available single-cell transcriptome sequencing data in post-GWAS analyses to further elucidate the genetic mechanisms of these traits. Finally, we performed genomic prediction within a mixed population to validate the MA-GWAMA signals.

## Methods

### Datasets

*Animals* A total of 3724 pigs from four breeds, Duroc (*N* = 1413), Landrace (*N* = 653), Yorkshire (*N* = 1156), and Piétrain (*N* = 502), were included in this study. The animals were born between 2017 and 2023 and raised in core breeding farms located in Anhui Province (*N* = 1014) and Guangxi Province (*N* = 2710), China. The pigs were housed at a stocking density of 15 pigs per pen and fed using an automatic feeding system that allowed ad libitum access to feed. They were managed under uniform conditions. The flooring of the pig house was slatted, allowing for easy waste disposal.

*Phenotypes* The pigs underwent routine performance testing at around 23 weeks of age (162 ± 3.78 days), where various traits were recorded, including body weight, body height, body length, number of teats, and backfat thickness. In this study, we focused specifically on carcass composition traits derived from CT scanning, which was performed when pigs approached a body weight of approximately 115 kg, following the procedure described by Pan et al. [12]. Prior to scanning, pigs were anesthetized via intramuscular injection of 0.1 mg/kg azaperone and 0.2 mg/kg ketamine. They were then positioned in a prone orientation on the CT bed, and whole-body scans were conducted to acquire 3D CT images. The 3D CT images were first used to count NR and then converted into a series of continuous 2D slices. Each CT slice had a thickness of approximately 5 mm and a resolution of 512 × 512 pixels. Before analysis, non-target structures such as the CT bed and visceral organs were removed. The images were then exported in DICOM format and converted to NIFTI files for subsequent processing. The intensity of each voxel was quantified on the Hounsfield scale and a deep neural network [12] was used to predict the lean meat, fat, and bone fractions on the basis of the voxel intensity in the slices. The body composition measures, including LMP and BP, were calculated by dividing the weight of each section by the whole-body weight. The outliers for each trait were identified using the interquartile range (IQR) method. Records below Q1-1.5*IQR or above Q3 + 1.5*IQR were removed, where Q1 is the 25% quantile, Q3 is the 75% quantile, and IQR = Q3-Q1. Based on phenotype filtering, 89 individuals were excluded, resulting in a total of 3635 individuals with complete records (1358 Duroc, 647 Landrace, 1132 Yorkshire, and 498 Piétrain pigs), which we termed ALL for brevity.

*Genotypes* Genomic DNA was extracted from the ear tissues of each individual using a routine phenol–chloroform extraction method [25]. The genomic data were then obtained by low-coverage whole-genome sequencing (lcWGS) via the DNBSEQ-T7 sequencer from BGI Genomics Co., Ltd. The raw sequencing reads were filtered using fastp v0.20.0 [26] with default filtering criteria to remove low-quality reads. GTX v2.1.5 [27] was used to align the clean reads to the reference genome of *Sus scrofa* 11.1 [28] to obtain clean BAM files. GLIMPSE2 [29] was then employed to correct genotypes with low depth and to impute missing genotypes based on the haplotype reference panel from PHARP [30]. A total of 41,828,284 SNPs were obtained following imputation, and SNPs with an information quality score (INFO SCORE) less than 0.3 were removed, leaving 41,727,553 SNPs.

*eQTL dataset* We downloaded the summary statistics of significant variant-gene associations for each tissue from the publicly available PigGTEx database (https://piggtex.farmgtex.org) [23] for subsequent colocalization and TWAS.

*scRNA-seq dataset* To explore gene expression at the single-cell level, we collected publicly available scRNA-seq datasets from multiple sources. Specifically, we obtained processed scRNA-seq data from the Pig Single Cell RNA Atlas (https://dreamapp.biomed.au.dk/pigatlas) [24], which provides a multiple-organ single-cell transcriptomic map containing 222,526 pig cells from 20 tissues and organs. We also downloaded annotated scRNA-seq data from Duroc pig muscle tissue via ALPHADB (https://alphaindex.zju.edu.cn/ALPHADB) [31]. In addition, we retrieved raw scRNA-seq data from primary human femoral head tissue from the GEO database (accession number GSE169396) [32]. Notably, the datasets from the Pig Single Cell RNA Atlas and ALPHADB are pre-annotated, whereas the GSE169396 dataset consists of unprocessed, raw matrices.

### Group division

**Fig. 1:**
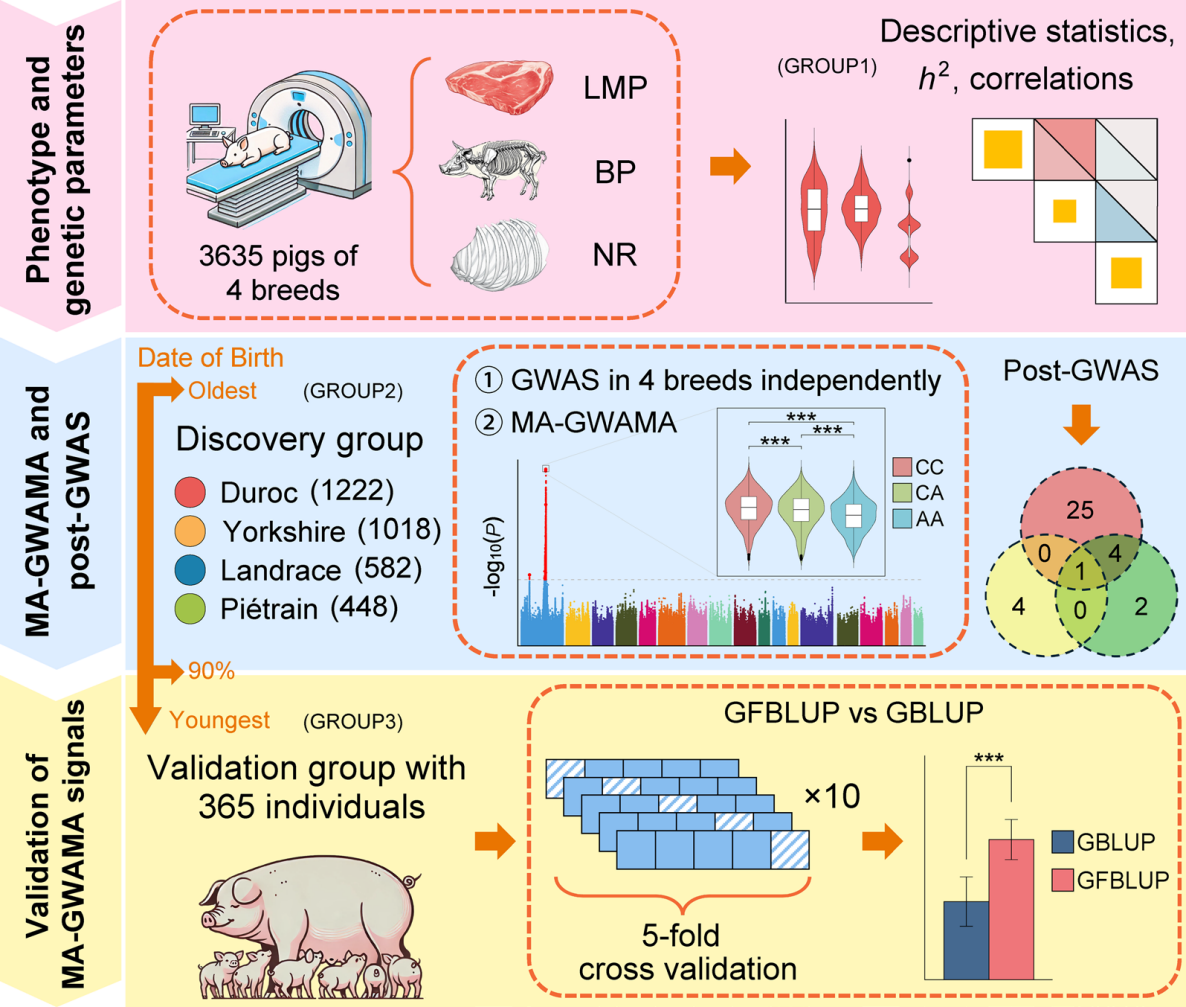
The main workflow used this study. Phenotypic and genomic data were collected from 3635 individuals, followed by breed-specific estimation of genetic parameters for three traits (pink section). Subsequently, a discovery group was formed within each breed by selecting the top 90% oldest individuals based on birth date. GWAS was performed separately within each breed, and a MA-GWAMA was then conducted. The results of MA-GWAMA were further subjected to functional annotation and enrichment analyses. Summary statistics from the MA-GWAMA were used for TWAS and colocalization analyses to identify significant genes and overlapping variants (blue section). Finally, a mixed dataset composed of the youngest 10% of individuals from each breed was used for genomic prediction to assess the effectiveness of incorporating significant loci identified by MA-GWAMA into genomic selection (yellow section)

Before downstream analysis, we defined the groups of animals used in each analysis (Table 1). The ALL group comprised the full dataset of 3635 individuals from four pig breeds after applying quality control based on the INFO SCORE. This dataset served as the starting point for deriving all subsequent analysis groups, namely, GROUP1, GROUP2, and GROUP3 (Table 1). GROUP1 encompassed the same individuals as ALL group but was partitioned into four breed-specific datasets. These subsets were used for estimating genetic parameters separately for each breed. The method to create GROUP2 and GROUP3 involved splitting animals from each breed into two subsets based on their birth dates. GROUP2 also referred to as the discovery group, comprised the oldest 90% of animals and was used for GWAS. GROUP3 also referred to as the validation group, comprised the youngest 10% of animals and was used to verify the effectiveness of the MA-GWAMA signals (Fig. 1). GROUP2 consisted of four separate breed-specific datasets, while GROUP3 was a combined dataset containing individuals from all four breeds. It is important to note that the Duroc population used in this study included two distinct breeding lines, referred to as S1 (*N* = 989) and S2 (*N* = 369). When dividing the Duroc animals into GROUP2 and GROUP3, birth dates were considered separately for S1 and S2 to ensure that individuals from both lines were represented in both groups. However, in all subsequent analyses, these two lines were treated as a single breed.

**Table 1:** Groups and information used in each analysis

Group	Breed	Sample size	Analysis	
ALL	Four breeds mixed	3635	Filter INFO SCORE and generate the following groups	
GROUP1	Duroc	1358	Genetic parameter estimation	
	Landrace	647	Genetic parameter estimation	
	Yorkshire	1132	Genetic parameter estimation	
	Piétrain	498	Genetic parameter estimation	
GROUP2 (Discovery group)	Duroc	1222	GWAS	Multi-ancestry meta-analysis
	Landrace	580	GWAS	
	Yorkshire	1018	GWAS	
	Piétrain	448	GWAS	
GROUP3 (Validation group)	Four breeds mixed	365	Validation of MA-GWAMA signals

Quality control was applied separately to the genomic data of each breed in GROUP1 and GROUP2. SNPs with minor allele frequencies (MAF) < 0.05 or deviating from Hardy–Weinberg equilibrium (HWE, *P* < 1 × 10^–10^) were removed. For GROUP3, the same quality control criteria were applied to the combined four-breed dataset.

### Genetic parameter estimation

Genetic parameters of the three traits were estimated for each breed in GROUP1, using the following multi-trait model:

1 $$\mathbf{y}=\mathbf{X}\mathbf{b}+\mathbf{Z}\mathbf{a}+\mathbf{e}$$

where $\mathbf{y}$ is the vector of phenotypic observations across the three traits; $\mathbf{b}$ is the vector of fixed effects, including sex (2 levels), farm & herd (27 levels), year & season (12 levels) of measurement; body weight, and the first five principal components (PC) calculated by PLINK v1.90 [33] using all SNPs were used as covariates; $\mathbf{X}$ and $\mathbf{Z}$ are incidence matrices relating the phenotypes to the fixed and random effects, respectively; and $\mathbf{a}$ is the vector of additive genetic random effects, assumed to follow a multivariate normal distribution *N*(0, **G**${\otimes} {{\varvec{\Sigma}}}_{{\varvec{a}}}$), where ${{\varvec{\Sigma}}}_{{\varvec{a}}}$ is the genetic (co)variance matrix, ${\otimes}$ is the Kronecker product of matrices, and **G** is the genomic relationship matrix (GRM) constructed using genotypes of the pruned SNPs (obtained by the command “–indep-pairwise 50 5 0.3” in PLINK v1.90 [33]) using HIBLUP v1.4.0 [34] based on the following formula:

2 $$\mathbf{G}=\frac{\mathbf{Z}{\mathbf{Z}}^{\text{T}}}{tr(\mathbf{Z}{\mathbf{Z}}^{\text{T}})/\text{n}}$$

where $\mathbf{Z}$ is the numeric coded *n* × *m* genotype matrix, where *n* is the number of individuals and *m* is the number of SNPs, and $tr$ is the trace of the matrix. The elements of the *i*^th^ column in the $\mathbf{Z}$ matrix were $2-2{p}_{A}$ for genotype *AA*, $1-2{p}_{A}$ for *Aa*, and $-2{p}_{A}$ for *aa*, where ${p}_{A}$ is the MAF at locus *i* [35]. Further, $\mathbf{e}$ is the vector of residual random effects following *N*(0, **I**${\otimes}{{\varvec{\Sigma}}}_{{\varvec{e}}}$), where **I** is an identity matrix and ${{\varvec{\Sigma}}}_{{\varvec{e}}}$ is the residual (co)variance matrix. The (co)variance matrices were as follows:

3 $${{\varvec{\Sigma}}}_{{\varvec{a}}}=\left[\begin{array}{cc}\begin{array}{cc}{\sigma }_{a1}^{2}& {\sigma }_{a1a2}\end{array}& {\sigma }_{a1a3}\\ \begin{array}{cc}\begin{array}{c}{\sigma }_{a2a1}\\ {\sigma }_{a3a1}\end{array}& \begin{array}{c}{\sigma }_{a2}^{2}\\ {\sigma }_{a3a2}\end{array}\end{array}& \begin{array}{c}{\sigma }_{a2a3}\\ {\sigma }_{a3}^{2}\end{array}\end{array}\right]$$

4 $${{\varvec{\Sigma}}}_{{\varvec{e}}}=\left[\begin{array}{cc}\begin{array}{cc}{\sigma }_{e1}^{2}& {\sigma }_{e1e2}\end{array}& {\sigma }_{e1e3}\\ \begin{array}{cc}\begin{array}{c}{\sigma }_{e2e1}\\ {\sigma }_{e3e1}\end{array}& \begin{array}{c}{\sigma }_{e2}^{2}\\ {\sigma }_{e3e2}\end{array}\end{array}& \begin{array}{c}{\sigma }_{e2e3}\\ {\sigma }_{e3}^{2}\end{array}\end{array}\right]$$

where ${\sigma }_{ai}^{2}$ and ${\sigma }_{ei}^{2}$ are the additive genetic variance and random residual variance for trait *i* (LMP, BP, and NR, respectively) and ${\sigma }_{st}$ represents the covariance between variables *s* and *t*. The variance components were estimated using the average information restricted maximum likelihood (AI-REML) algorithm implemented in HIBLUP v1.4.0 [34]. The heritability for each trait (${h}^{2}$) was estimated using the following formula:

5 $${h}^{2}={\widehat{\sigma }}_{a}^{2}/{\widehat{\sigma }}_{p}^{2}$$

where ${\widehat{\sigma }}_{p}^{2}$ is the estimate of the phenotypic variance of the trait. The equations for estimating genetic correlations (${r}_{g}$) and phenotypic correlations (${r}_{p}$) are as follows [36]:

6 $${r}_{g}=\frac{{\widehat{\sigma }}_{{a}_{m}{a}_{n}}}{\sqrt{{\widehat{\sigma }}_{{a}_{m}}^{2} {\widehat{\sigma }}_{{a}_{n}}^{2}}}; {r}_{p}=\frac{{\widehat{\sigma }}_{{a}_{m}{a}_{n}}+{\widehat{\sigma }}_{{e}_{m}{e}_{n}}}{\sqrt{\left({\widehat{\sigma }}_{{a}_{m}}^{2}+{\widehat{\sigma }}_{{e}_{m}}^{2}\right)\left({\widehat{\sigma }}_{{a}_{n}}^{2}+{\widehat{\sigma }}_{{e}_{n}}^{2}\right)}}$$

where *m* and *n* are two distinct traits.        

### GWAS and MA-GWAMA

For each trait, phenotypes corrected for fixed effects from model (1) were extracted using the *lm* function in R. GWAS were then performed separately for the corrected phenotypes of each of the four breeds based on GROUP2, using the following univariate linear mixed model implemented in GEMMA v0.98.5 [37]:

7 $${\mathbf{y}}^{\boldsymbol{*}}=1\alpha +\mathbf{x}\beta +\mathbf{u}+\mathbf{e}$$

where ${\mathbf{y}}^{\boldsymbol{*}}$ is a vector of corrected phenotypic values for a given trait; *α* is the overall population mean; $\beta$ is an estimate of the allele substitution effect for the evaluated SNP; $1$ is a vector of 1 s; $\mathbf{x}$ is a vector of SNP genotype codes, as used to compute **G**; **u** is a vector of random additive genetic effects, assumed to follow a normal distribution *N*(0, **G**${\sigma }_{a}^{2}$), where **G** is the GRM constructed using GCTA v1.92.4 [38], consistent with model (1), and ${\sigma }_{a}^{2}$ is the additive genetic variance. The residual errors (**e**) follow a normal distribution *N*(0, **I**${\sigma }_{e}^{2}$), where **I** is an identity matrix, and ${\sigma }_{e}^{2}$ is the random residual variance.

To improve power to detect trait-associated variants and identify genomic loci shared across breeds, we performed a multi-ancestry meta-analysis of summary statistics obtained from the GWAS of each of the four breeds using the METAL software [39]. We set the genome-wide significance threshold for the *P-*value to 5 × 10^–8^. The proportion of variance in the phenotype explained (PVE) by a given SNP, was calculated following Shim et al. [40] as:

8 $$PVE=\frac{2{\widehat{\beta }}^{2}{p}_{A}(1-{p}_{A})}{2{\widehat{\beta }}^{2}{p}_{A}\left(1-{p}_{A}\right)+{\left(se(\widehat{\beta })\right)}^{2}2n\cdot {p}_{A}(1-{p}_{A})}$$

where $\widehat{\beta }$ and $se(\widehat{\beta })$ are the effect size and standard error of the effect size of each SNP estimated by MA-GWAMA, respectively; where ${p}_{A}$ is the MAF of the SNP, and *n* is the sample size. Manhattan and quantile–quantile plots were drawn using the CMplot v4.3.3 [41] package in R.

### SNP annotation and gene enrichment

Gene annotations were performed for regions within 0.5 megabases (Mb) upstream and downstream of genome-wide significant SNPs using the GALLO v1.3 R package [42] based on the pig gene transfer format files (GTF, Release 111). For these annotated genes, we conducted Gene Ontology (GO) annotation and Kyoto Encyclopedia of Genes and Genomes (KEGG) pathway enrichment analysis using the clusterProfiler v4.10.1 [43] package in R. To simplify the GO enrichment results, the rrvgo package [44] in R was used to calculate semantic similarity between genes and group similar terms to reduce redundancy in the GO sets. To identify independent significant signals accurately, we used the conditional and joint association analysis (COJO) method [45] implemented in GCTA v1.92.4 [38] to select lead SNPs (r^2^ < 0.1) [46] from among the genome-wide significant SNPs. The gene closest to each lead SNP based on the reference genome was identified as a candidate gene. To understand the functions of the candidate genes, we manually queried PubMed (https://pubmed.ncbi.nlm.nih.gov), PigGTEx (https://piggtex.farmgtex.org) [23], and GeneCards (https://www.genecards.org) to obtain information on their associations with the traits studied.

### Colocalization analyses of eQTLs and TWASs

We performed colocalization analysis to investigate whether the genetic variants identified in MA-GWAMA and for gene expression traits (*cis*-eQTLs) share the same causal variant, thereby linking genes expressed in specific tissues to genetic loci associated with traits of interest. To achieve this, we utilized public gene expression data from PigGTEx (https://piggtex.farmgtex.org), which provides comprehensive expression profiles across porcine tissues. For each significant MA-GWAMA signal, we identified all expressed genes (eGenes) located within a 0.5 Mb window upstream and downstream of the lead SNP [47]. Using the R package coloc v5.2.3 [48], we calculated the posterior probabilities (PP) for five hypotheses (*H0* to *H4*) that describe the relationship between the MA-GWAMA and eQTL signals. Specifically:

*H0*: Neither the MA-GWAMA nor the eQTL signal exist.

*H1*: Only the MA-GWAMA signal exists.

*H2*: Only the eQTL signal exists.

*H3*: Both signals exist but they do not share a causal variant.

*H4*: Both signals exist and they share the same causal variant.

A colocalization signal was considered significant if the posterior probability of *H4* (PP.H4) was greater than 0.8. This approach provides insights into the biological mechanisms underlying MA-GWAMA loci by linking them to gene expression in relevant tissues.

To identify genes whose *cis*-regulated expression is associated with carcass composition traits, TWASs were conducted across 34 tissues using summary statistics from MA-GWAMA and the elastic net method on the FarmGTEx TWAS-Server (https://twas.farmgtex.org) [49]. Briefly, the MA-GWAMA summary statistics file for each trait was uploaded to the server, including columns for the chromosome, position, name, minor and major allele of the SNP, the *P*-value obtained from the MA-GWAMA, and the effect size of the minor allele. The expression levels of genetically regulated genes were then quantitatively predicted. Significant trait-tissue-gene associations were defined as those with a *P-*value less than 0.05/t, where t is the number of genes tested in a TWAS.

### Single-cell data processing and enrichment analysis

For the raw human bone scRNA-seq data from GEO (accession number GSE169396), we performed subsequent analyses using Scanpy v1.9.2 [50]. In total, we obtained 21,323 cells from bone tissue through the following steps: (1) Low-quality cells were filtered (*pct_counts_mt* < 10, 200 < *n_genes_by_counts* < 7500) and doublets were removed using Scrublet [51]. (2) The data were normalized and highly variable genes were selected using default parameters. (3) The expression data were scaled for PC analysis and the first 10 PCs were applied to perform uniform manifold approximation and projection (UMAP). (4) The UMAP were clustered using the Leiden algorithm and the clusters were annotated with marker genes referenced in Bandyopadhyay et al. [52] and CellMarker 2.0 [53]. (5) Orthologous gene mapping was performed based on *Sus scrofa* 11.1 Ensembl (v108) annotation and only one-to-one orthologous genes between pig and human were retained to ensure cross-species comparability. Annotation details for all cells are provided in Additional file 1: Table S1.

To investigate the relationships between genes and traits at the single-cell level, we used MA-GWAMA summary statistics to identify potential trait-associated genes via MAGMA v1.10 [54]. Each candidate gene was assigned a weight based on its MA-GWAMA-derived MAGMA z-score and was inversely adjusted for its gene-specific technical noise level in all annotated single cell datasets. Next, we calculated trait-relevant and control scores using the transcriptional profiles of individual cells with scDRS v1.0.3 [55] and derived the corresponding P-values from these scores. These scores were then used in downstream analyses to establish associations between cell types and traits and to examine co-expression between prioritized trait-relevant genes and those implicated by MA-GWAMA.

### Validation of the MA-GWAMA signals

To validate the identified variants from MA-GWAMA, we implemented a genomic prediction analysis, using the individuals in GROUP3 as the validation set. The prediction accuracy was evaluated via fivefold cross-validation. Briefly, the animals in GROUP3 were randomly divided into five equal subsets. In each fold, all individuals’ genotypic data and the phenotypic records of four subsets (training set, *N* = 292) were used to predict the estimated breeding values (EBVs) of individuals in the remaining subset (validation set, *N* = 73). The prediction accuracy was quantified by calculating the Pearson’s correlation coefficient between the corrected phenotypic values and the EBVs. The corrected phenotypic values were obtained by fitting a single-trait genomic best linear unbiased prediction (GBLUP) model to the genotypic and phenotypic data of GROUP3 using HIBLUP v1.4.0 [34] to adjust for fixed effects and covariates. Two methods were used to estimate EBVs. The first was the single-trait GBLUP model, which constructs a GRM using all SNPs from GROUP3. The second was the single-trait genomic feature best linear unbiased prediction (GFBLUP) model, which incorporates prior knowledge by partitioning SNPs into feature and background sets, as described by Sarup et al. [56]:

9 $$\mathbf{y}=\mathbf{X}\mathbf{b}+{\mathbf{Z}}_{\mathbf{f}}\mathbf{f}+{\mathbf{Z}}_{\mathbf{r}}\mathbf{r}+\mathbf{e}$$

For each trait, significant SNPs identified from MA-GWAMA were intersected with SNPs in GROUP3 to define the feature set (2008 SNPs for LMP, 15 for BP, and 11,755 for NR). The remaining SNPs constituted the background set (583,480 for LMP, 582,953 for BP, and 582,953 for NR). In Eq. (9), $\mathbf{f}$ and $\mathbf{r}$ are vectors of additive genetic effects contributed by SNPs in the feature and background sets, respectively, and are assumed to follow independent normal distributions: $\mathbf{f}$~*N*(**0**, **G**_**f**_${\sigma }_{{a}_{f}}^{2}$), $\mathbf{r}$~*N*(0, **G**_**r**_${\sigma }_{{a}_{r}}^{2}$). The GRMs **G**_**f**_ and **G**_**r**_ were constructed from these SNP subsets using HIBLUP v1.4.0 [34] based on Eq. (2), and ${\sigma }_{{a}_{f}}^{2}$ and ${\sigma }_{{a}_{r}}^{2}$ are their respective additive genetic variances. The matrices ${\mathbf{Z}}_{\mathbf{f}}$ and ${\mathbf{Z}}_{\mathbf{r}}$ are design matrices that link observations to EBVs, and other symbols are analogous to those in Eq. (1) but represent their single-trait model equivalents in this context. The entire process was repeated 10 times. A t-test was employed to ascertain whether there was a discernible disparity between the outcomes yielded by the two methods. As a control, we also performed ten rounds of random selection across the whole genome, selecting the same number of SNPs as the GWAS signals to serve as feature SNPs. Within each round, ten replicates of five-fold cross-validation were conducted, and the final prediction accuracy of the control was defined as the average value across the ten rounds.

## Results

### Genotype imputation and population structure

**Fig. 2:**
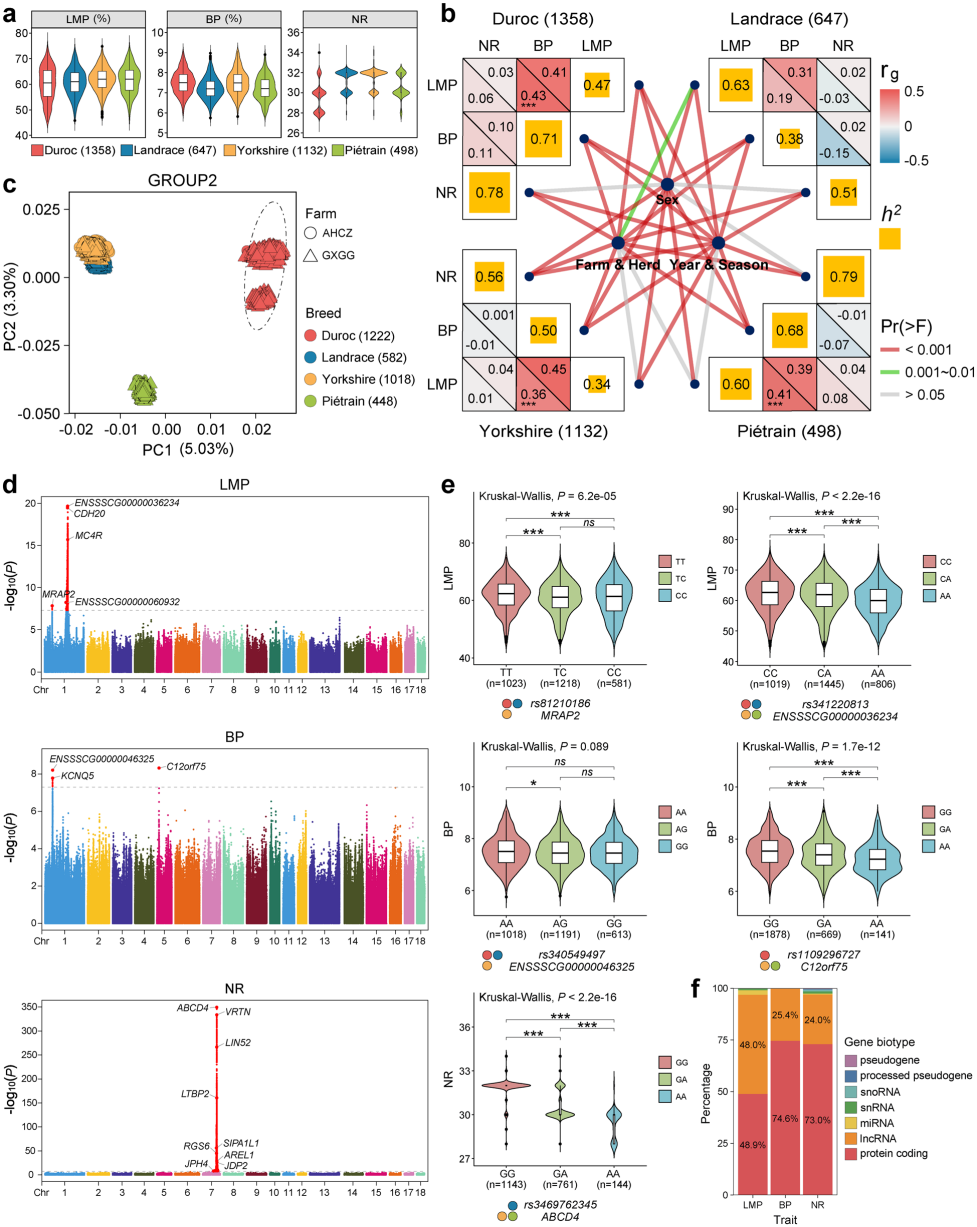
Phenotypic statistics, estimates of genetic parameters, and MA-GWAMA results for the evaluated carcass composition traits. **a** Violin plots showing the phenotypic distribution of three traits across the four breeds. **b** Results of genetic parameter estimation. Both the x- and y-axes represent the traits, with the x-axis at the top and the y-axis on the left. The size and values within the yellow squares indicate the heritability of each trait, while the color and values within the triangles represent the correlations between traits. The upper triangles indicate phenotypic correlations, and the lower triangles represent genetic correlations, with * denoting significance based on the chi-square test. The color of the line connecting the two blue points in the middle indicates the significance of the fixed effect on the trait, and the trait is represented by the coordinates on the left. **c** Population structure plot for GROUP2, with dashed lines representing the 95% confidence ellipses. The numbers in brackets to the right of the breeds indicate the number of individuals, and the numbers in brackets on the axes indicate the percentage explained by the principal components. **d** Manhattan plots of the MA-GWAMA results for three traits. The X-axis represents the chromosomes, and the Y-axis represents the -log_10_ (*P*) value. SNPs that passed the genome-wide significance threshold (*P* < 5 × 10^–8^) are highlighted in red. **e** Distribution of phenotypic values for different genotypes of lead SNPs. * indicates significant differences between two groups based on t-tests, and *ns* indicates that the difference in the t-test is not significant. The circles to the left of the rs IDs represent the populations in which the loci were detected, with colors corresponding to the populations shown in **(c)**. **f** Bar plot showing the percentage of gene biotypes annotated from significant SNPs

The imputation results from lcWGS are presented in Additional file 2: Figure S1. The PHARP [30] dataset included 597 Duroc, 142 Landrace, 401 Yorkshire, and 35 Piétrain pigs, none of which were part of our study. The average coverage of the genomic data was 0.66 ± 0.11 (see Additional file 2: Figure S1a), with most sequencing depths ranging from 2 × to 3 × (see Additional file 2: Figure S1a). After filtering by the INFO SCORE and MAF, 97% of the SNPs used for GWAS had imputation accuracies greater than 0.9 (see Additional file 2: Figure S1a). The SNP density map (see Additional file 2: Figure S1b) indicates that the whole genome information was represented by the imputed SNP data. Figure 2c shows the population structure based on the first and second PCs, with individuals from Landrace, Yorkshire, and Piétrain pigs clustering separately. The Duroc pigs were completely distinguished from the other three breeds based on the first PC.

### Phenotypic descriptive statistics and genetic parameters

The majority of individuals had LMP phenotypes between 50 and 70% (Fig. 2a). The Duroc and Yorkshire breeds presented relatively high and comparable BP, whereas the Landrace and Piétrain displayed lower and similar BP values. The Duroc breed exhibited three density peaks in NR, at 28, 30, and 32 ribs, whereas the other three breeds showed only two peaks, at 30 and 32 ribs (Fig. 2a). Estimates of the phenotypic and genetic correlations of the same pair of traits were similar across breeds, while estimates of heritability and correlations between different traits showed great differences between the four breeds (Fig. 2b). For example, the heritability estimates of LMP ranged from 0.38 to 0.71 from Landrace, Yorkshire, Piétrain, and Duroc. Estimates of genetic correlations between LMP and BP were low to moderate positive across all four breeds (0.19 to 0.43), whereas NR was estimated to have little to no genetic correlation with the other two traits (−0.15 to 0.11).

### Genetic loci of carcass composition traits

The quantile–quantile plots of the MA-GWAMA results are shown in Additional file 2: Figure S1c and the lead SNP information obtained from the COJO analysis in Table 2. A total of 2025 SNPs across two major regions on chromosome 1 exhibited significant associations with LMP (Fig. 2d) and these SNPs annotated protein-coding genes and lncRNAs in similar proportions (Fig. 2f). Notably, a large cluster of these SNPs resided within a 9.5 Mb region on *Sus scrofa* chromosome (SSC) 1. The most significant SNP on SSC1 (rs341220813, SSC1:160,499,409) explained 2.6% of the phenotypic variance for LMP (*P* = 2.17 × 10^–20^) and was positioned 0.13 Mb upstream of the *ENSSSCG00000036234* gene. At this SNP, individuals with the *CC* genotype exhibited significantly greater LMP values (4.0%) than those with the *AA* genotype (Fig. 2e). Another common significant SNP on SSC1, rs81210186 (SSC1:53290945) is located in the intronic region of the *MRAP2* gene.

**Table 2:** MA-GWAMA^a^ lead SNPs obtained from COJO^b^ analysis for the evaluated carcass composition traits at a suggestive significance (*P* < 5 × 10^–8^) level

Trait	Lead SNP	Nearest genes	Chromosome	Position (bp)	Allele1	Allele2	Allele1 frequency	N^c^	Beta (se)^d^	PVE (%)^e^	*P*-value
LMP^f^	rs341220813	*ENSSSCG00000036234*	1	160,499,409	A	C	0.478	3270	-2.12 (0.23)	2.55	2.17 × 10^–20^
	rs328396302	*ENSSSCG00000060932*	1	148,869,509	A	G	0.642	3270	-1.43 (0.25)	1.03	5.80 × 10^–9^
	rs81210186	*MRAP2*	1	53,290,945	T	C	0.570	2822	1.50 (0.26)	1.13	1.46 × 10^–8^
BP^g^	rs1109296727	*C12orf75*	5	14,725,729	A	G	0.204	2688	-0.12 (0.02)	1.26	4.74 × 10^–9^
	rs340549497	*ENSSSCG00000046325*	1	52,583,860	A	G	0.556	2822	0.11 (0.02)	1.19	6.14 × 10^–9^
NR^h^	rs3469762345	*ABCD4*	7	97,595,573	A	G	0.269	2048	-1.17 (0.03)	43.94	4.37 × 10^–350^
	rs332899302	*SIPA1L1*	7	128,249,358	T	G	0.525	2688	-0.67 (0.04)	8.58	6.44 × 10^–57^
	rs1112293562	*RGS6*	7	95,895,992	A	G	0.282	2048	-0.59 (0.04)	8.95	1.08 × 10^–45^
	rs341974796	*AREL1*	7	97,872,854	T	C	0.680	2688	-0.46 (0.04)	4.53	1.18 × 10^–29^
	rs3471072556	*JDP2*	7	98,536,806	T	C	0.590	1670	0.46 (0.04)	6.47	6.12 × 10^–27^
	rs691610562	*JPH4*	7	75,486,361	A	G	0.211	3270	0.29 (0.05)	1.02	6.35 × 10^–9^

There were 17 significant SNPs associated with BP, on SSC1 and SSC5 (Fig. 2d), most of which were annotated to reside in protein-coding genes, and the rest were lncRNAs (Fig. 2f). The most significant SNP for BP was rs1109296727 (*P* = 4.74 × 10^–9^, SSC5:14725729), which was positioned 5.6 Kb upstream of the *C12orf75* gene and explained 1.3% of the phenotypic variation (Table 2). At this SNP, individuals with the *GG* genotype exhibited significantly higher BP values (4.4%) than those with the *AA* genotype (Fig. 2e). The lead SNP on SSC1, rs340549497 (*P* = 6.14 × 10^–9^, SSC1:52583860) was positioned at 52.58 Mb and explained 1.2% of the phenotypic variation (Table 2).

A total of 19,003 significant SNPs associated with NR were identified on SSC7 (Fig. 2d). This trait exhibited the greatest number of SNPs that reached genome-wide significance among the three analyzed traits. Notably, NR displayed a distinct genomic region composed of significant SNPs concentrated on SSC7. The gene biotypes (e.g., protein-coding genes, lncRNAs, miRNAs) annotated from these SNPs were similar to those of the BP trait (Fig. 2f). The most significant SNP was rs3469762345 (*P* = 4.37 × 10^–350^, SSC7:97595573), which was positioned 9.9 Kb downstream of the *ABCD4* gene and accounted for 43.9% of the phenotypic variation (Table 2). At this SNP, the average phenotypes for NR of individuals with different genotypes varied greatly (Fig. 2e), and the 0.5 Mb genomic region near it contains a multitude of previously reported positional candidate genes for NR in pigs (Fig. 2d).

### Functional enrichment of annotated genes

**Fig. 3:**
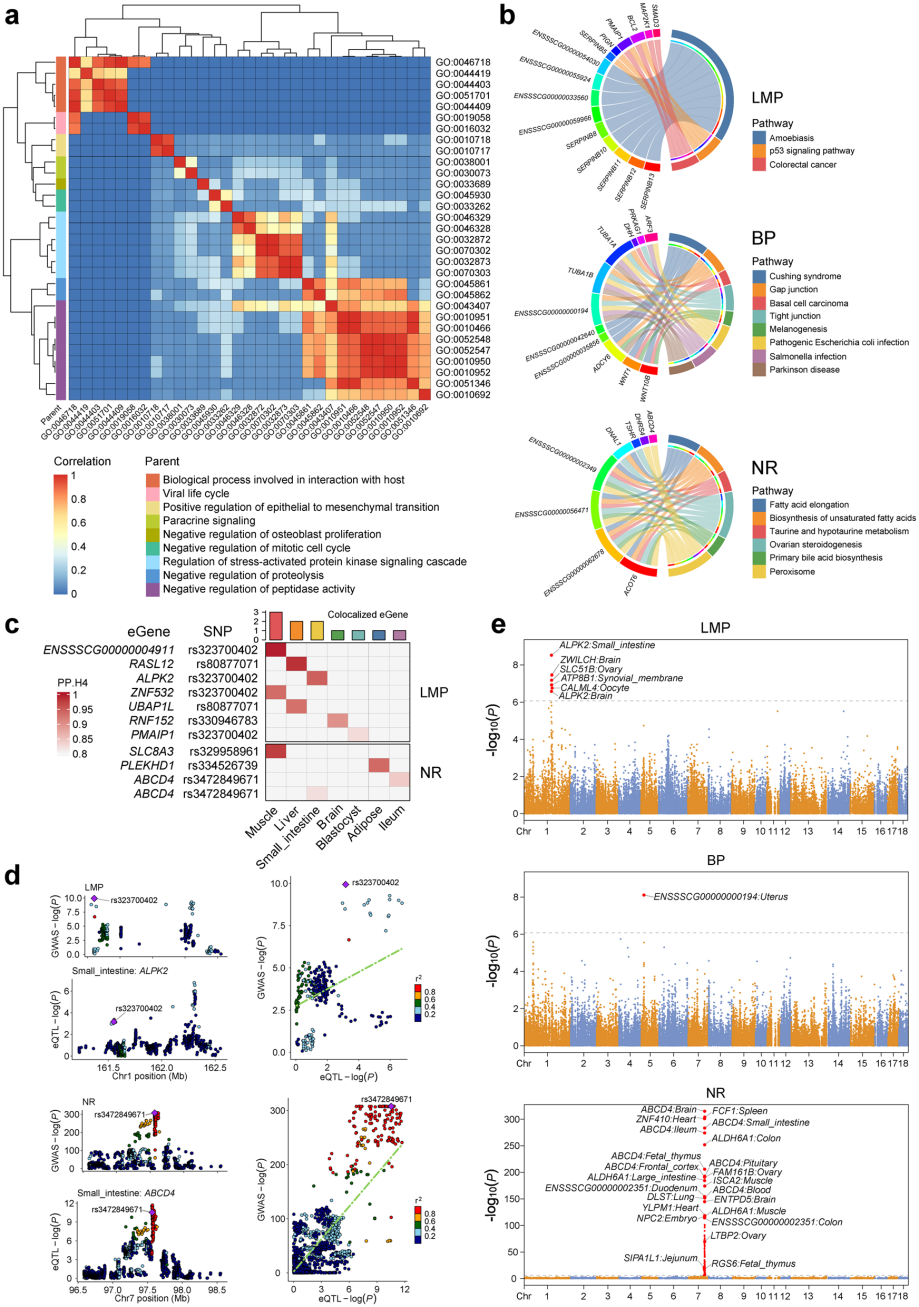
Results from gene enrichment, colocalization, and TWAS analysis. **a** Heatmap of the similarity matrix for GO terms across all traits. Each cell represents the semantic similarity score between two GO terms, with hierarchical clustering used to group similar terms. **b** KEGG pathway enrichment chord plots for three traits. The left half of each plot represents genes, while the right half represents significantly enriched KEGG pathways. The connections indicate associations between genes and pathways. **c** Heatmap of colocalization results for gene-SNP pairs with a PP.H4 greater than 0.8 for each trait. The rows report the overlap for individual gene and SNP pairs, and the columns report the PP.H4 score in each tissue. The color of each square is based on the code found to the left of each confidence level, with darker colors indicating greater confidence that the same variant influences both the phenotype and gene expression of the trait. The gray cells indicate that the gene is not an eQTL target in that tissue. The bar plot above the heatmap displays the number of colocalized eGenes with high confidence (PP.H4 > 0.8) in each tissue. **d** Locus zoom plots comparing candidate gene loci in MA-GWAMA of each trait with eQTL loci. The top-left plot represents the Manhattan plot for the MA-GWAMA within a specific gene region, while the bottom-left plot represents the Manhattan plot for the corresponding eQTLs. The purple diamonds represent the top SNPs from the colocalization analysis, while the remaining dots, in various colors, represent different levels of linkage disequilibrium with the top SNPs. **e** Manhattan plots of the TWAS results across all traits. The X-axis represents chromosomes, and the Y-axis represents the -log_10_ (*P*) value. Gene tissue pairs with *P*-value less than 0.05/n, where n is the number of genes tested in the TWAS, are highlighted in red

To further explore the biological functions and pathways of the genes, which were annotated from the significant SNPs identified in the MA-GWAMA, we performed GO and KEGG enrichment analyses. Genes associated with LMP were significantly enriched for numerous GO terms related to protein synthesis and metabolism (see Additional file 3: Table S2), such as the regulation of endopeptidase activity (GO:0052548) and enzyme inhibitor activity (GO:0004857). Genes associated with BP were enriched predominantly for GO terms related to cellular components, such as cytoplasmic microtubule (GO:0005881). Genes associated with NR were enriched for several molecular functions associated with enzyme activity, such as thiolester hydrolase activity (GO:0016790) and CoA hydrolase activity (GO:0016289). The heatmap in Fig. 3a illustrates the semantic similarity among all significantly enriched GO terms. These GO terms are involved primarily in biological processes involved in interaction with host, regulation of stress-activated protein kinase signaling cascade, and negative regulation of peptidase activity. Among the KEGG enrichment results, the p53 signaling pathway (map04115) was identified as potentially associated with LMP (Fig. 3b). Genes associated with BP were enriched for pathways associated with cell junctions, including gap junction (map04540) and tight junction (map04530). Genes associated with NR were enriched for multiple biosynthesis-related pathways, such as biosynthesis of unsaturated fatty acids (map01040), ovarian steroidogenesis (map04913), and primary bile acid biosynthesis (map00120).

### eQTL colocalization and TWAS

Colocalization analysis identified eQTLs for 10 genes across 7 tissues that colocalized with two traits, LMP and NR, forming 11 tissue-gene-trait pairs (Fig. 3c). The gene *ENSSSCGO0000004911* in muscle was the eGene with the highest PP.H4 for LMP. Although this gene was not annotated in the MA-GWAMA results, its SNP rs323700402 (*P* = 1.16 × 10^–10^, SSC1:161551900) was significant. This SNP also serves as an eQTL for *ALPK2*, *ZNF532*, and *PMAIP1*, all of which were annotated in MA-GWAMA. Notably, the *ABCD4* gene colocalized with the MA-GWAMA results for both intestinal tissues, suggesting a potential role of the intestine in NR regulation.

A MA-GWAMA summary-based TWAS across 34 tissues using the ElasticNet TWAS pipeline identified 20, 17, and 151 candidate genes for LMP, BP, and NR, respectively (see Additional file 4: Table S3). The expression levels of 28, 13, 13, 12, 11, and 10 genes in muscle, adipose, small intestine, brain, lung, and liver tissues, respectively, were significantly correlated with LMP, BP, and NR (False Discovery Rate, FDR < 0.05). Notably, expression of the *ALPK2* gene not only showed significant correlation with LMP for both tissues, but also significantly correlated with MA-GWAMA and colocalization results. Among the three traits, NR had the greatest number of significantly associated genes that aligned with MA-GWAMA results (Fig. 3e). The level of expression of *ABCD4* again demonstrated a significant association with NR, which was consistent with the findings from MA-GWAMA. This gene is expressed in multiple tissues, including the brain, small intestine, ileum, and blood, with the most significant associations observed for brain.

### Gene-based analysis and enrichment at the single-cell level

**Fig. 4:**
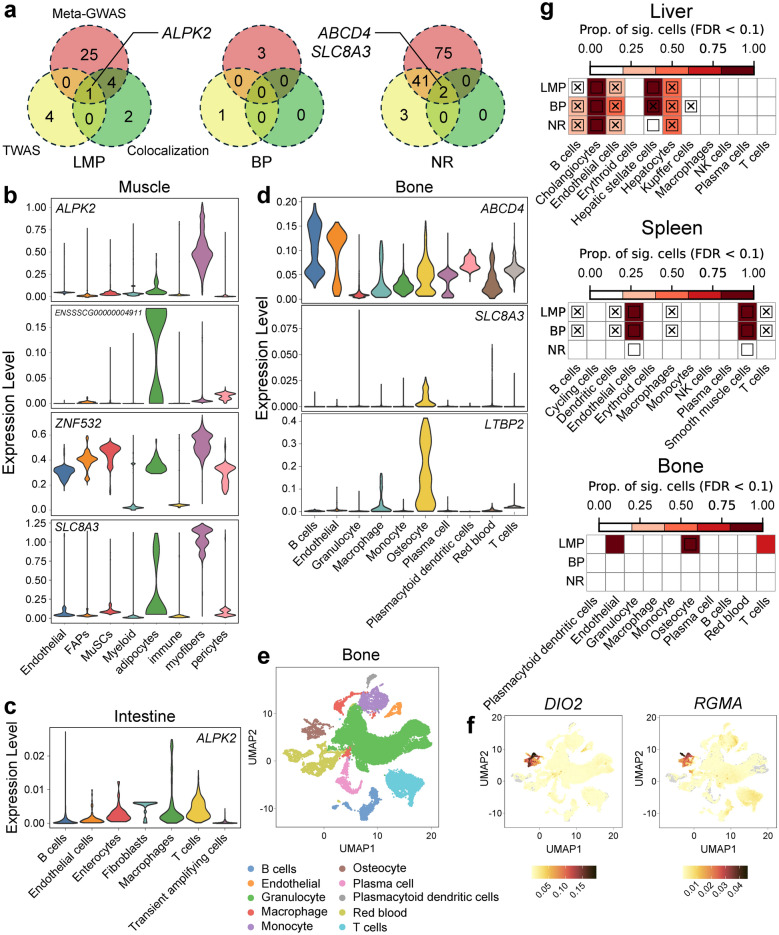
Gene-based single-cell enrichment results. **a** Venn diagrams illustrating the number of genes identified for the three traits through MA-GWAMA, TWAS, and colocalization analysis. Red represents MA-GWAMA-identified genes, yellow represents TWAS-identified genes, and green represents colocalized genes. **b** Violin plots showing the expression levels of selected genes across different cell types in muscle. **c** Violin plots depicting the expression levels of *ALPK2* across various cell types in intestinal tissue. **d** Violin plots illustrating the expression levels of genes such as *ABCD4* across different cell types in bone. **e** UMAP plot displaying distinct cell populations in bone. **f** UMAP plots showing the expression patterns of *DIO2* and *RGMA* in bone. **g** Heatmaps illustrating trait-associated cell type enrichment in liver, spleen, and bone tissues. The color gradient represents the proportion of significantly associated cells within each cell type. Squares indicate significant associations between specific cell types and traits (FDR < 0.1). Cross symbols denote significant heterogeneity in trait associations among individual cells within a specific cell type

Based on the results of MA-GWAMA, TWAS, and colocalization analyses, we identified genes that were significant for each of these three analyses (Fig. 4a). To investigate their cell-specific expression patterns, we utilized publicly available scRNA-seq data from 20 pig tissues (see Additional file 2: Figure S1d), as well as scRNA-seq data from muscle tissues (see Additional file 2: Figure S1e) that were accumulated in our laboratory. In muscle, the *ALPK2* gene is expressed predominantly in myofibers (Fig. 4b). The genes *ENSSSCG00000004911* and *ZNF532*, which were significantly colocalized in muscle (Fig. 3d), exhibited distinct expression patterns: *ENSSSCG00000004911* was expressed mainly in adipocytes, whereas *ZNF532* was broadly expressed across various cell types (Fig. 4b). Among the genes that colocalized with NR in muscle, *SLC8A3* was expressed primarily in myofibers and adipocytes (Fig. 4b).

Because *ALPK2* was found to be associated with the small intestine in both the TWAS and colocalization analyses, we examined its expression across different intestinal cell types. As shown in Fig. 4c, *ALPK2* was widely expressed across all cell types in the intestinal. In bone, we identified 10 cell types (Fig. 4e) by performing homologous gene conversion using human femoral head scRNA-seq data. The *ABCD4* gene was expressed in all bone tissue cell types, whereas *SLC8A3* was almost exclusively expressed in osteocytes (Fig. 4d). Among all genes expressed in osteocytes, *LTBP2* had the highest expression level (Fig. 4d). Additionally, we observed elevated expression levels of genes such as *DIO2* and *RGMA* in osteocytes (Fig. 4f).

To further elucidate the cellular mechanisms underlying carcass composition traits, we conducted cell-type-specific enrichment analysis, which revealed intriguing associations between different cell types and these traits. In the liver, most cell types presented significant associations with LMP and BP (Fig. 4g). In the spleen, endothelial cells and smooth muscle cells were also significantly associated with both these traits. In bone, endothelial cells, osteocytes, and T cells were significantly associated with only LMP (Fig. 4g). Additionally, significant enrichment results were observed in cells from tissues such as the lung, kidney, and occipital lobe (see Additional file 2: Figure S1f).

### Prediction performance of the MA-GWAMA signals

**Fig. 5:**
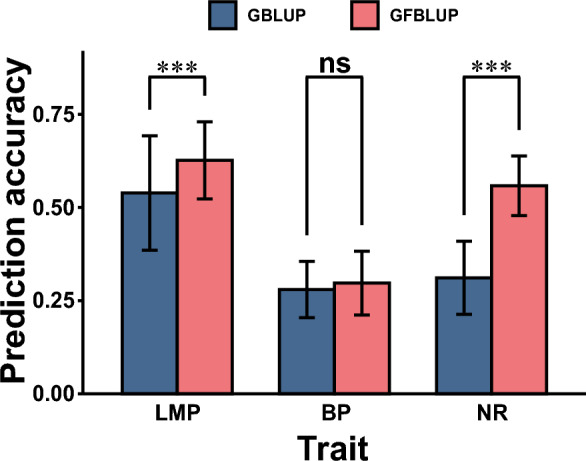
Prediction accuracies for the evaluated traits using MA-GWAMA signals with GBLUP and GFBLUP models. The X-axis represents the trait, and the Y-axis represents the correlation coefficient between the GEBV and the corrected phenotype. *** indicates that the *P*-value of the t-test is less than 0.001, and *ns* indicates that the difference in the t-test is not significant

The prediction accuracy reflects the effectiveness of the variants identified from MA-GWAMA. The GBLUP model demonstrated prediction accuracies of 0.54 for LMP, 0.28 for BP, and 0.31 for NR (Fig. 5). SNP screening of MA-GWAMA results was performed based on the genome-wide significance threshold, and the resulting prediction accuracies of GFBLUP for the three traits increased to 0.63, 0.30, and 0.56 (Fig. 5), respectively, i.e. by 16.3% for LMP, by 6.1% for BP, and by 79.4% for NR (see Additional file 5: Table S4). The GFBLUP results based on randomly selected SNPs showed no significant improvement over GBLUP for BP and NR, but a significant increase in prediction accuracy was observed for LMP (see Additional file 2: Figure S1g). We further examined the variance components estimated for the feature SNPs for LMP and found that the MA-GWAMA signals explained a significantly larger proportion of variance than the randomly selected SNPs (see Additional file 2: Figure S1h).

## Discussion

In this study, we analyzed phenotypic data on carcass composition traits in pigs obtained through CT measurements collected between 2017 and 2023. CT offers several advantages for assessing these traits. It provides non-invasive, high-resolution imaging that enables accurate and consistent measurements. Additionally, we utilized lc-WGS data, which offers a cost-effective alternative to conventional WGS while providing higher marker density and improved imputation accuracy compared to SNP chip-based genotyping. The higher marker density of lc-WGS enhances the ability to detect novel and rare variants that may be missed by SNP chips [57]. Furthermore, the imputation process yielded a high INFO SCORE (Fig. S1a), indicating reliable genotype calls. However, compared with conventional WGS, lc-WGS relies on accurate reference panels for imputation, which may introduce biases in populations with limited reference data [58]. Despite this limitation, our lcWGS dataset exhibited sufficient coverage and a well-defined population structure, providing a solid foundation for identifying genomic regions and SNPs associated with carcass composition traits in pigs.

The heritability estimates observed in this study varied considerably among breeds (Fig. 2b), indicating that breed plays a crucial role in the variation in these traits. The predominantly moderate to high heritability values suggest that these traits are substantially influenced by genetic factors (Fig. 2b). In our study, the estimated heritability of LMP in Duroc pigs was lower than the 0.73 reported by Cabling et al. [59], who analyzed 690 purebred Duroc pigs at slaughter. Similarly, the heritability estimate for LMP in Yorkshire pigs was lower than those reported by Ducos et al. [60] and Deru et al. [61]. Ducos et al. [60] examined 3039 Large White pigs at slaughter and reported a heritability of 0.60 for estimated carcass lean content (%), while Deru et al. [61] analyzed 880 Large White pigs fed traditional grain-based diets before slaughter and reported a heritability of 0.62. Additionally, Ducos et al. [60] reported a heritability of 0.68 in 2695 French Landrace pigs, which is comparable to our estimate of 0.63. Previous studies have reported moderate to high heritability for NR in pigs [62, 63], with our study estimating heritabilities ranging from 0.51 to 0.79 (Fig. 2b), which aligns with these findings. However, there is limited research on the genetic parameters of BP, and our results may serve as a reference for future studies. Although BP and NR are related to bone, they exhibit virtually no correlation (Fig. 2b). We speculate that this lack of correlation arises because BP is a ratio-based phenotype, meaning that an increase in NR does not affect the proportion of bone in carcass weight. This is likely because pigs with more ribs are also longer, with a corresponding increase in muscle and fat.

The *ENSSSCG00000036234* gene was the most significant gene identified in our MA-GWAMA results for LMP (Fig. 2d). A recent phenome-wide association study (pheWAS) conducted by Zeng et al. [21] reported a significant association of *ENSSSCG00000036234* with meat and carcass traits, including backfat thickness (*P* = 2.05 × 10^–12^) and loin muscle depth (*P* = 7.86 × 10^–19^). Similarly, Cai et al. [64] performed a large-scale association study on daily weight gain in pigs and identified *ENSSSCG00000036234* as a candidate gene for average daily weight gain. They also highlighted the *MRAP2* gene, which was also detected in our MA-GWAMA results (Fig. 2d). Other significant SNPs near the *ENSSSCG00000036234* region were mapped to various genes, including *MC4R* (Fig. 2d), which has been previously associated with average daily gain, feed conversion ratio, and ham weight in Italian Large White and Duroc pigs [65]. The *MC4R* gene may also influence the deposition of lean meat and fat [65]. In previous studies on pigs, no reports were found linking *ENSSSCG00000046325* or *C12orf75* to carcass traits.

Among the candidate genes for NR, Mikawa et al. [66] were the first to identify and characterize *VRTN*, a gene with DNA-binding activity that exhibits a strong association with vertebral number in pigs. Zhang et al. [67] conducted a GWAS in a Large White × Minzhu intercross pig population and pinpointed the locus for rib number to a 2.38 Mb region on SSC7, further identifying *LTBP2* as a novel candidate gene for NR in pigs. Additionally, *ABCD4* has been proposed as a key candidate gene for NR in Beijing Black pigs [68]. The most significant SNP mapped to *ABCD4* was significantly associated with pig teat number (*P* = 1.29 × 10^–14^) and carcass length (*P* = 3.11 × 10^–14^) in a pheWAS conducted by Zeng et al. [21].

Our GO and KEGG enrichment analyses provide a comprehensive understanding of the biological functions and pathways associated with carcass composition traits in pigs. These findings highlighted the importance of protein synthesis, metabolism, and signaling pathways in shaping these traits. The integration of eQTL colocalization and TWAS analyses identified several genes and their causal SNPs associated with the studied carcass composition traits at the transcriptional level, further narrowing the scope of candidate genes.

The *ALPK2* gene showed significant associations in the MA-GWAMA, TWAS, and colocalization analyses for LMP (Fig. 4a), providing strong evidence for its role as a candidate gene for this trait. Park [69] reported that *ALPK2* plays critical roles in cardiogenesis and is specifically expressed in muscle, including the longissimus dorsi muscle. Notably, *ALPK2* was upregulated in the longissimus dorsi muscle of Wannanhua pigs compared with Yorkshire pigs, suggesting its involvement in muscle development and growth performance. The level of expression of this gene in brain and intestinal tissues was significantly correlated with LMP and strongly colocalized in intestinal tissues (Fig. 3c), which may be linked to the brain-gut axis. We hypothesize that the gut microbiota may influence LMP by regulating energy absorption and fat storage, making this a promising direction for future research. Additionally, eQTLs with pleiotropic effects on gene expression, such as rs323700402 (SSC1:161551900) (Fig. 3c), are likely to be true causal loci for the LMP trait.

It is noteworthy that many SNPs strongly associated with phenotypes in MA-GWAMA were not classified as eQTLs in any tissue. This discrepancy may be attributed to the systematic differences in hits between GWAS and *cis*-eQTL analyses, with eQTLs explaining only a small proportion of GWAS signals [70]. Another possible explanation is that our CT-based measurements of carcass composition traits revealed novel genetic signals not included in PigGTEx, preventing colocalization results from being obtained. According to the TWAS results, most candidate genes identified in muscle, adipose, small intestine, brain, lung, and liver tissues were closely associated with carcass composition traits (Fig. 3e), which aligns with our colocalization results.

Teng et al. [23] reported that *ABCD4* was the top associated gene in the small intestine TWAS for average backfat thickness and was also significantly associated with backfat thickness in brain. Their GWAS loci for backfat thickness colocalized with *cis*-eQTLs of *ABCD4* in both the brain and small intestine [23]. These findings suggest that *ABCD4* may be expressed in various tissues and is widely involved in regulating various biological processes.

Our single-cell analyses provided novel insights into cell type-specific contributions to carcass composition traits. The *ALPK2* gene is highly expressed in myofibers of muscle tissue (Fig. 4b), suggesting that it may regulate LMP in pigs through its activity in these cells. The *ENSSSCG00000004911* gene may function in adipocytes (Fig. 4b) and was also identified in a meta-GWAS of the fatness trait in pigs by Zeng et al. [13]. In bone, the *ABCD4* gene is highly expressed, whereas genes such as *SLC8A3* and *LTBP2*, which are highly expressed only in osteocytes, are more likely to be potential candidate genes for NR (Fig. 4d). Notably, *LTBP2* has previously been proposed as a candidate gene for NR in pigs [67]. Our single-cell analysis provides further support for this notion and suggests that *LTBP2* may play a specific role in NR determination through its function in osteocytes (Fig. 4d).

We found no significant associations between different cell types and carcass traits in muscle and adipose tissues. In contrast, different cell types in liver and spleen tissues (Fig. 4g), which may play a more active role in carcass composition than previously thought, showed significant associations. These findings enhance our understanding of the cellular mechanisms underlying these complex traits and highlight the potential for targeting specific cell types to improve genetic selection in pig meat production.

In this study we used a multi-breed population (GROUP3) to validate the identified signals via genomic prediction, because these signals were originally detected through the MA-GWAMA that combined results from the four breeds represented in GROUP2. The prediction accuracy of the GFBLUP model for carcass composition traits met our expectations, indicating that the SNPs identified through MA-GWAMA for CT-based traits are reliable. Although the randomly selected SNPs also showed an apparent improvement in prediction accuracy for LMP, the variance components associated with these SNPs were estimated to be nearly zero, suggesting that these loci contributed little to the phenotype. In contrast, the significant signals identified by MA-GWAMA explained a substantially larger proportion of variance and largely overlapped with previously reported loci [21, 64, 65], further supporting their biological relevance and validity. This comparison demonstrates that the improved prediction observed for features identified by MA-GWAMA is not a mere consequence of the GFBLUP model structure, but reflects the true informativeness of the identified signals.

The improvement in prediction accuracy for BP using the GFBLUP model compared to the GBLUP model was not statistically significant (*P* value of the t test > 0.05). This may be because the proportion of QTLs in the preselected genomic feature SNPs is very low, rendering the use of these preselected SNPs ineffective [71]. A study by Song et al. [72] on fish found that a GFBLUP model that incorporated GWAS results exhibited an average prediction accuracy for disease resistance and growth traits that was 6.2% lower than that of the GBLUP model. The genetic architecture of different traits may vary, affecting the precision of genomic predictions even when the same models and methods are applied [73]. In our study, predictions were based solely on SNP data, yet additional genomic factors, such as insertions, deletions, and structural variants, may also affect BP. Furthermore, there are only a few multi-omics studies for this trait, and identifying more effective variants through the integration of prior multi-omics data remains an ongoing challenge.

One limitation of our study is the relatively small dataset for CT-based phenotypes compared with traits that are easier and less expensive to measure. This may be one reason why causal loci could not be identified for BP. Additionally, because of the absence of gene expression data for bone tissue in the current PigGTEx database, we were unable to determine whether eQTLs associated with pig carcass composition traits exist in bone tissue. To address this gap, future studies should incorporate transcriptome analysis of bone tissue to enhance our understanding of gene expression patterns in this tissue.

A further limitation of our study is that the genomic prediction used to validate the effectiveness of identified variants relied on a multi-breed cohort due to insufficient individual breed sample sizes (particularly for underrepresented breeds like Piétrain). Although this design reduces breed-specific accuracy and departs from industry practice, which typically relies on within-breed models that account for distinct genetic architectures and selection objectives, it is adequate for this study because the primary purpose of genomic prediction here was to validate the MA-GWAMA signals. Nevertheless, our results underscore the critical need for expanded breed-specific datasets to enhance the practical applicability of genomic prediction. Moving forward, we aim to optimize the measurement and calculation of carcass composition traits, particularly BP, and refine the identification of causal variants by integrating comprehensive phenotypic, genotypic, and histological data.

## Conclusions

This study integrated multi-ancestry meta-analysis of GWASs with gene expression and scRNA-seq data to identify candidate genes for LMP, BP, and NR, as measured by CT in four pig breeds (Duroc, Landrace, Yorkshire, and Piétrain). *ALPK2* and rs323700402 (SSC1:161551900) were identified as the most likely candidate gene and causal variant for a LMP QTL. Additionally, *ABCD4* and *SLC8A3* demonstrated strong associations with NR across the three analytical approaches, while *LTBP2* may play an important role in NR at the cellular level. Incorporating multi-ancestry genome-wide association meta-analysis results as prior information led to improvements in genomic prediction accuracy for LMP, BP, and NR by 16.3%, 6.1%, and 79.4%, respectively. These findings highlight the utility of computed tomography-measured phenotypes in revealing the genetic basis of pig carcass composition traits.

## Supplementary Information

Additional file 1: Table S1. Cell types and their marker genes in bone

Additional file 2: Figure S1. Genotype data quality, quantile-quantile plots of MA-GWAMA, and scRNA-seq data analysis results. **(a)** Density distribution of sequencing coverage and depth for lcWGS data. Density distribution of INFO SCORE for imputed SNPs in lcWGS data. Density distribution of INFO SCORE after filtering out SNPs with MAF < 0.05. **(b)** Density distribution of retained SNPs after filtering out those with MAF < 0.05 and HWE violations (*P* < 1 × 10^−10^). The X-axis represents the physical positions on the chromosomes, while the Y-axis represents each autosome. The number of SNPs per 1 Mb window is indicated by the color scale at the bottom. **(c)** Quantile-quantile plots of MA-GWAMA results for three traits. The lambda (λ) value in the top left corner represents the genomic inflation factor. **(d)** UMAP plot identifying 20 tissues from publicly available scRNA-seq data. **(e)** UMAP plot displaying distinct cell populations in muscle. **(f)** Heatmaps illustrating trait-associated cell type enrichment in lung, kidney, and occipital lobe tissues. The color gradient represents the proportion of significantly associated cells within each cell type. Squares indicate significant associations between specific cell types and traits (FDR < 0.1). Cross symbols denote significant heterogeneity in trait associations among individual cells within a specific cell type. **(g)** Prediction accuracies for three traits using MA-GWAMA signals and randomly selected SNPs in GBLUP and GFBLUP models. The X-axis represents the trait, and the Y-axis represents the prediction accuracy, measured as the correlation coefficient between the GEBV and the corrected phenotypes. The models include GBLUP (all SNPs), GFBLUP using MA-GWAMA signals as feature SNPs, and GFBLUP using randomly selected SNPs as feature SNPs. *** indicates that the *P*-value of the t-test is less than 0.001, and *ns* indicates a non-significant difference. **(h)** Variance components explained by MA-GWAMA signals and randomly selected SNPs in GFBLUP for LMP. The X-axis represents the type of feature SNPs used in GFBLUP, and the Y-axis represents the variance components explained by the feature SNP set. Each point denotes a fold from the cross-validation analysis (n = 48 for MA-GWAMA signals and n = 500 for random SNPs). Due to non-convergence in two folds when using MA-GWAMA signals as features, only 48 results were available. *** indicates that the *P*-value of the t-test is less than 0.001.

Additional file 3: Table S2. Significant GO and KEGG enrichment results (FDR < 0.05) for LMP, BP, and NR

Additional file 4: Table S3. Significant TWAS results (FDR < 0.05) for three traits

Additional file 5: Table S4. Prediction accuracies and standard deviations from ten replicates/rounds of genomic prediction for three traits using MA-GWAMA signals and randomly selected SNPs

## Data Availability

The phenotypic and genotypic data used in this study were obtained from the core farms of commercial companies and are not publicly available, but are available from the corresponding author upon reasonable request. The pig summary statistics of significant variant-gene associations for each tissue were obtained from [23]. The scRNA-seq data from the Pig Single Cell RNA Atlas were obtained from [24], and the Duroc pig muscle scRNA-seq expression matrix was obtained from [31]. The scRNA-seq data of primary human femoral head tissue were obtained from [32].
