# Supplementary figures and images for: Multi-ancestry genome-wide association meta-analysis identifies candidate genes for computed tomography-based carcass composition traits in pigs
Source: Genet Sel Evol. 2025 Dec 17;58:5. doi: 10.1186/s12711-025-01023-8 (PMC12784493; doi:10.1186/s12711-025-01023-8)

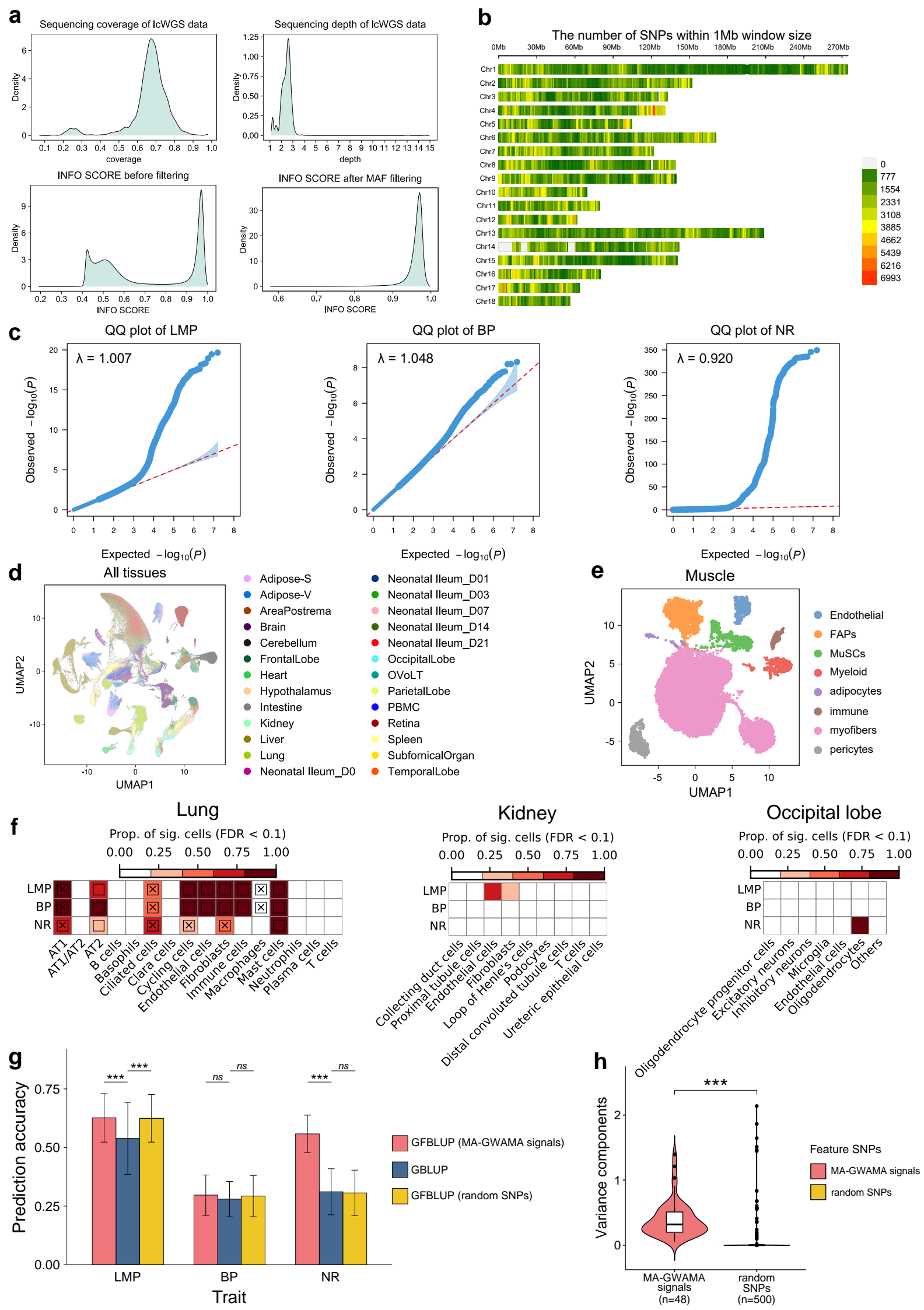

Supplement: Supplementary file 2 — Additional file 2: Figure S1. Genotype data quality, quantile-quantile plots of MA-GWAMA, and scRNA-seq data analysis results. (a) Density distribution of sequencing coverage and depth for lcWGS data. Density distribution of INFO SCORE for imputed SNPs in lcWGS data. Density distribution of INFO SCORE after filtering out SNPs with MAF < 0.05. (b) Density distribution of retained SNPs after filtering out those with MAF < 0.05 and HWE violations (P < 1 × 10^−10^). The X-axis represents the physical positions on the chromosomes, while the Y-axis represents each autosome. The number of SNPs per 1 Mb window is indicated by the color scale at the bottom. (c) Quantile-quantile plots of MA-GWAMA results for three traits. The lambda (λ) value in the top left corner represents the genomic inflation factor. (d) UMAP plot identifying 20 tissues from publicly available scRNA-seq data. (e) UMAP plot displaying distinct cell populations in muscle. (f) Heatmaps illustrating trait-associated cell type enrichment in lung, kidney, and occipital lobe tissues. The color gradient represents the proportion of significantly associated cells within each cell type. Squares indicate significant associations between specific cell types and traits (FDR < 0.1). Cross symbols denote significant heterogeneity in trait associations among individual cells within a specific cell type. (g) Prediction accuracies for three traits using MA-GWAMA signals and randomly selected SNPs in GBLUP and GFBLUP models. The X-axis represents the trait, and the Y-axis represents the prediction accuracy, measured as the correlation coefficient between the GEBV and the corrected phenotypes. The models include GBLUP (all SNPs), GFBLUP using MA-GWAMA signals as feature SNPs, and GFBLUP using randomly selected SNPs as feature SNPs. *** indicates that the P-value of the t-test is less than 0.001, and ns indicates a non-significant difference. (h) Variance components explained by MA-GWAMA signals and randomly selected SNPs in [file 12711_2025_1023_MOESM2_ESM.pdf]
